# Vegetation change impacts on soil organic carbon chemical composition in subtropical forests

**DOI:** 10.1038/srep29607

**Published:** 2016-07-11

**Authors:** Xiaoping Guo, Miaojing Meng, Jinchi Zhang, Han Y. H. Chen

**Affiliations:** 1 Collaborative Innovation Center of Sustainable Forestry in Southern China of Jiangsu Province, Nanjing Forestry University, 159 Longpan Road, Nanjing, Jiangsu 210037, China; 2 Faculty of Natural Resource Management, Lakehead University, 955 Oliver Road, Thunder Bay, Ontario P7B 5E1, Canada

## Abstract

Changes in the chemical composition of soil organic carbon (SOC) might strongly affect the global carbon cycle as it controls the SOC decomposition rate. Vegetation change associated with long-term land use changes is known to strongly impact the chemical composition of SOC; however, data on the impacts of vegetation change following disturbance events of short durations and succession that occur frequently in forest ecosystems via diverse management objectives on SOC chemical composition are negligible. Here we examined the impacts of vegetation changes on the chemical composition of SOC by sampling soils of native broad-leaved forests, planted mixed broad-leaved and coniferous forests, and tea gardens in eastern China. We used nuclear magnetic resonance spectroscopy to quantify SOC chemical composition. We found that among all components of SOC chemical composition, alkyl carbon (C) and aryl C were more liable to change with vegetation than other SOC components. Soil pH was negatively correlated to the relative abundances of alkyl C and N-alkyl C, and Shannon’s index of overstory plant species was positively correlated to the relative abundances of phenolic C and aromaticity. Our results suggest that vegetation changes following short disturbance events and succession may strongly alter SOC chemical composition in forest ecosystems.

Globally, soil organic carbon (SOC) amounts to approximately 2,344 Gt, making it the largest terrestrial carbon (C) pool^1^,^2^,^3^. Even small changes in SOC pool decomposition might make significant changes in the atmospheric carbon (C) concentration^4^, and could lead to a strong positive feedback to climate change^5^. SOC decomposition rates are strongly associated with its chemical composition^6^, with decomposition rates decreasing from O-alkyl C to alkyl C^7^,^8^. Thus, shifts in SOC chemical composition affect SOC stabilization^9^,^10^.

The input of C to the soil profile is determined by C allocation, decomposition, and production^11^. Vegetation change associated with long-term land use changes may result in SOC chemical compositional shifts^12^,^13^,^14^. For example, when natural forests were converted to hoop pine plantations, O-alkyl C decreased and alkyl C increased^13^. After native shrub lands were converted to chestnut plantations, both alkyl C and O-alkyl C decreased^14^. Vegetation change and the corresponding management may alter soil aggregates, which influence SOC chemical composition^6^. It also leads to lignin variation, which consequently impacts the yield of lignin-derived phenols and carboxyl^15^. Vegetation change affects lignin degradation through alterations in soil texture^11^, and increased yields of phenolic CuO oxidation products^15^. The soil texture consequently affects carbohydrates^16^, and O-alkyl C (mainly carbohydrate) was found be predominate in the larger fractions, whereas alkyl carbon (primarily polymethylene) dominated the clay fractions^17^. The influence of vegetation change and the corresponding management toward the initiation of changes in lignin and carbohydrates might be attributed to particular microbes^18^,^19^. As a result of the different growth characteristics and nutrient demands of specific microbial groups, the changes in lignin and carbohydrates may favor the growth of certain microbial groups over others^20^. Variations in SOC chemical composition associated with vegetation change might be attributed to the different patterns of mineralization of plant-derived microbial carbohydrate inputs^15^,^21^. However, it remains unclear whether vegetation change following disturbance events of short durations, which frequently occur naturally or via diverse management objectives in forest ecosystems, affects the chemical composition of SOC.

We sampled forest soils from native broadleaf forests, mixed evergreen broadleaved and coniferous forests, and tea gardens in Fengyang Mountain nature reserve, Zhejiang Province, China, to examine the impact of vegetation change following disturbance events of short durations and succession on the chemical composition of SOC. The mixed broadleaved and coniferous forests, and tea gardens were converted from native broadleaf forests during 1971–1973, and since 1975, the study forests have been protected from human disturbances. We hypothesized more similar SOC chemical compositions between native broadleaf forests and broadleaf tea gardens than those between native broadleaf forests and mixed broadleaved and coniferous forests, as plants play an important role in SOC dynamics^22^, mainly by means of litter quality^23^, which changes in the quantity and type of fresh organic matter that enters the soil^1^; thus, manipulating the SOC chemical composition accordingly^21^. We alternatively hypothesized that the conversion from native broadleaf forests to tea gardens has greater impacts on the chemical composition of SOC than that the conversion to mixed forests, as the conversion to tea garden involved complete clearance, which was a more intensive disturbance than the conversion from native forests to mixed forests via selective deforestation. We also hypothesized that overstory plant diversity, as well as soil physicochemical characteristics, may be attributable to the variation in SOC chemical composition. We addressed: (i) impacts of vegetation change from native broadleaved to mixed forests and tea garden on SOC chemical composition, and (ii) associations between tree species diversity, soil physicochemical characteristics and SOC chemical composition.

## Results

### Vegetation change impacts on tree diversity and soil physicochemical characteristics

In this study, when evergreen broadleaved forests were converted to mixed forests and tea gardens, the Shannon index decreased significantly (Table 1). Soil physicochemical characteristics varied among vegetation types, primarily in bulk density and total phosphorus concentration. Bulk density significantly decreased from broadleaved forests to tea gardens, while total phosphorus concentration strongly decreased from broadleaved forests to mixed forests. The impacts of vegetation change on pH, total organic carbon, and total nitrogen concentration were less apparent (Table 1).

### Vegetation change impacts on SOC chemical composition

**Figure 1:**
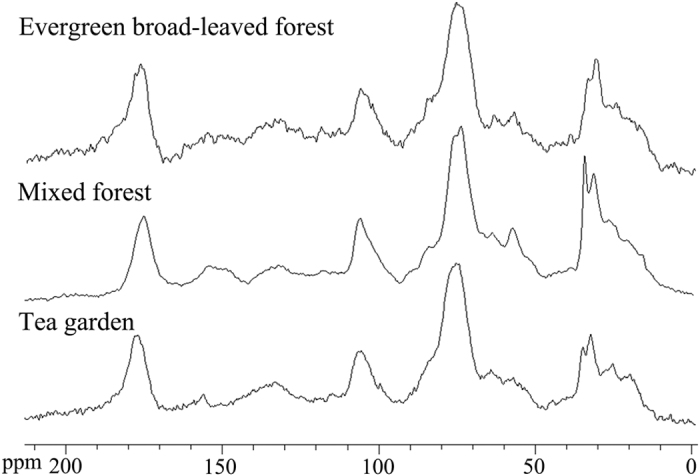
Solid-state ^13^C NMR spectra for soil organic carbon (SOC) in three vegetation types.

**Figure 2:**
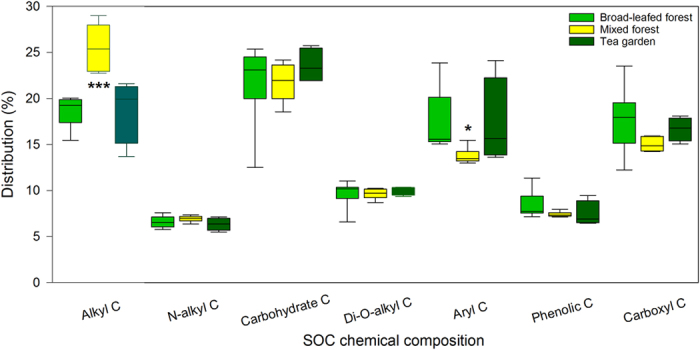
The chemical composition of soil organic carbon in three vegetation types. For each chemical shift range, different letters refer to a significant difference (*p* < 0.05).

**Figure 3:**
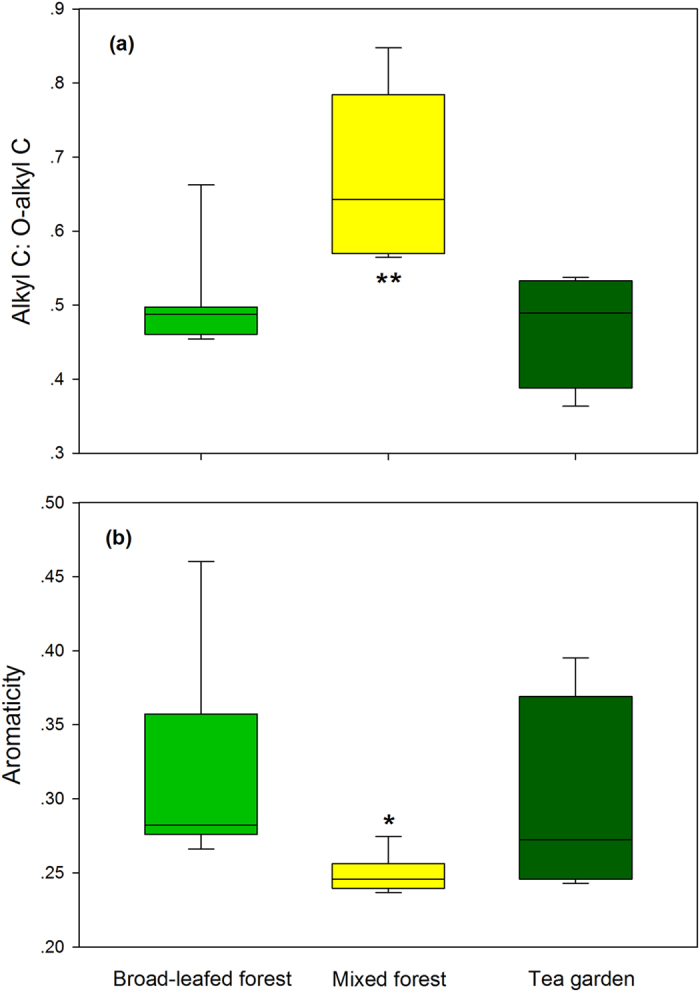
Ratio of alkyl C to O-alkyl C and the aromaticity in three vegetation types . For each index, different letters refer to a significant difference (*p* < 0.05).

The integration of the major regions of ^13^C resonance revealed that alkyl C and O-alkyl C (C_0−45 ppm_ and C_45−110 ppm_, respectively) were the dominant C components in all soils (Fig. 1). A multiple-response permutation procedures analysis (MRPP) showed the differences of SOC chemical composition among vegetation types with *P* = 0.003 (Table 2). The relative abundance of alkyl C and aryl C differed significantly among three vegetation types, with the highest relative abundance of alkyl C and lowest aryl C in mixed forests (Fig. 2, Table 3). The A/O-A also differed significantly among vegetation types, with the highest one in mixed forests, while the aromaticity in the mixed forest was lower than the other forest types (Fig. 3a,b, Table 3). However, there was no significant difference in SOC chemical composition between broadleaved forests and tea gardens. For the other components of SOC chemical composition, there were no significant differences among three vegetation types (Fig. 2, Table 3).

**Figure 4:**
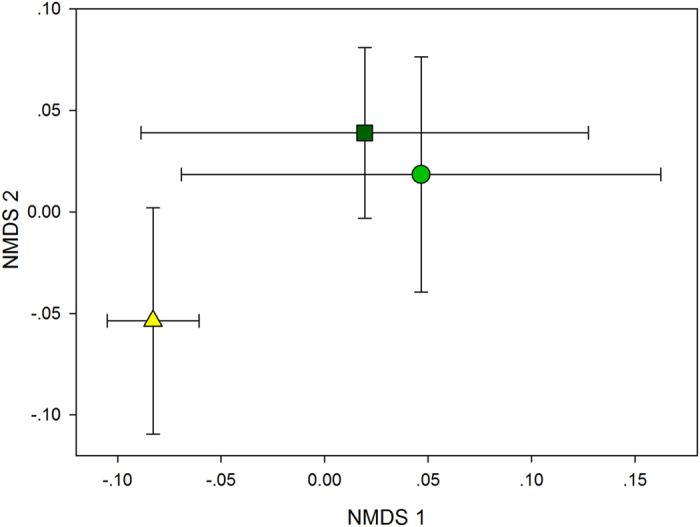
Non-metric multi-dimensional scaling ordination of three vegetation types. Site scores represent soil organic carbon chemical composition assemblages. The large symbols represent the centroids of all samples from three vegetation types, and lines indicate the SE along each NMDS axis. Circles represent native broad-leaved forests; triangles represent mixed forests, and squares represent tea gardens.

**Figure 5:**
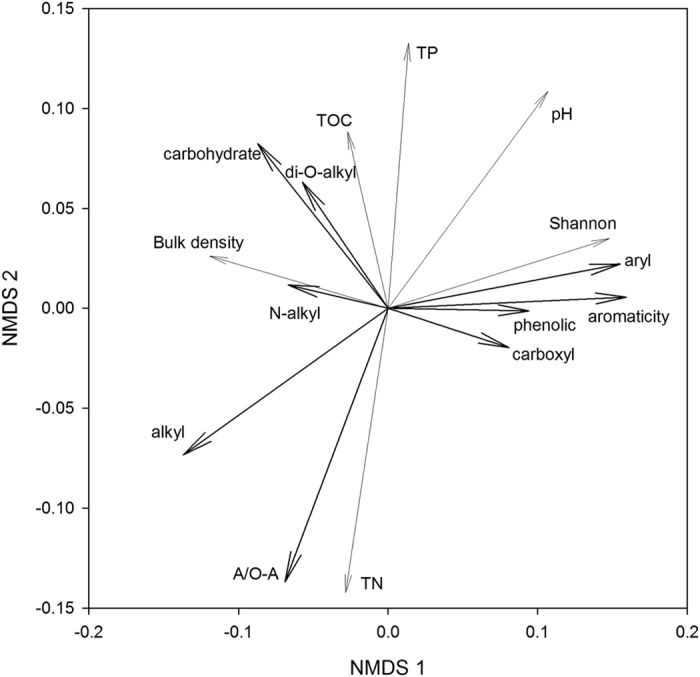
Non-metric multi-dimensional scaling ordination of soil organic carbon chemical composition in three vegetation types. Black lines with long arrows indicate soil organic carbon chemical composition. Grey lines with short arrows indicate tree diversity and soil physicochemical characteristics. Shannon – Shannon index.

The NMDS ordination resulted in a final stress of 0.046 (Fig. 4). The distance between broadleaved forest and mixed forest was considerable, and depicted along the NMDS axis 1 and axis 2 with *P* = 0.020 and *P* = 0.023, respectively. However, the distance between tea garden and broadleaved forest was not distinct along either the NMDS axis 1 or axis 2 (*P* = 0.640 and *P* = 0.541, separately). The chemical composition of SOC assemblages was strongly associated with all individual components, particularly along the NMDS axis 1 (Table 4). However, the chemical composition of SOC assemblages had statistically weak association with forest and soil properties. The NMDS axis 1 corresponded mainly to a gradient of increasing Shannon index and pH (*P* = 0.059 and *P* = 0.098, respectively). The NMDS axis 2 corresponded primarily to a gradient of increasing total soil phosphorus (TP) and decreasing total soil nitrogen (TN) (*P* = 0.085 and *P* = 0.104, respectively) (Fig. 5, Table 5).

### Influence of soil physicochemical characteristics and plant species diversity on SOC chemical composition

Soil pH was negatively correlated with alkyl C and N-alkyl C, with *P* = 0.043 and *P* = 0.039, respectively. The Shannon index was positively correlated with phenolic C, and the aromaticity (*P* = 0.018 and 0.050, respectively). Soil physicochemical characteristics and Shannon index did not significantly correlate to the other components of chemical composition (*P* > 0.05) (Table 6).

## Discussion

Vegetation controls the magnitude of SOC stocks as well as the composition of SOC in soils^24^, and is thus regarded as one of critical factors in SOC composition^25^. We found that the differences in the chemical composition of SOC are primarily represented by alkyl C and aryl C, which is similar to previous findings^24^,^26^,^27^,^28^. This indicated that alkyl C and aryl C were more sensitive to vegetation changes.

As we anticipated, the conversion of subtropical native broadleaved evergreen forest to other vegetation types impacted the chemical composition of SOC, where the impact differed between vegetation types. We hypothesized that the conversion of native broadleaved evergreen forests to tea garden may result in stronger alterations in SOC chemical composition. It was considered that the disturbance of soil aggregates was much more potent during the conversion to tea gardens than the conversion to mixed forests, which might significantly impact the chemical composition of SOC^6^. Contrary to our hypothesis, the difference of SOC chemical composition in the conversion to mixed forests was more distinct.

This may be partially attributable to the different biochemical content (e.g., lignin or carbohydrates) in the litter of pine needles in the support of its preferred microbial community, as trees can alter soil properties through root-microbe interactions^13^. This resulted in a distinct difference of the chemical composition of SOC in mixed forests^29^. Smith, *et al*.^30^ deemed that the effects of vegetation or land use changes on C content of soil is tree species-dependent. That the conversion of native broadleaved forests to tea gardens resulted in no significant difference in the chemical composition of SOC may be attributed to the less differences in the organic carbon chemical composition in leaves or roots between broadleaved trees^13^. Collectively, these findings indicate that the vegetation type is a critical factor that influences SOC chemical composition.

The physiochemical characteristics of the soil may favor certain microbial groups over others, thereby leading to shifts in the composition of microbial communities^20^,^31^. In this study, alkyl C was found to be significantly correlated with soil pH, and this may be attributed to the microbial community composition of different pH optima^32^, which controlled SOC formation processes accordingly^21^. As we anticipated, tree species diversity contributed to SOC chemical composition. The impact of plant diversity on SOC decomposition is chiefly through influential physicochemical and biological pathways^2^. Briefly, plant diversity may influence the quantity and quality of organic carbon inputs from litterfall and root decomposition, as well as microclimates, to subsequently alter physicochemical characteristics of the soil^6^,^33^,^34^,^35^. Subsequently, the belowground soil microbial biomass and microbial activities are stimulated^36^, leading to differences in SOC composition.

Our study highlights that vegetation change is a critical factor that impacts the SOC chemical composition in forests ecosystems, where the impacts were found to be variable from native broadleaved forests to other vegetation types following disturbances of short durations and natural succession. The impact of conversion from broadleaved forests to mixed forests on the chemical composition of SOC was stronger than the conversion to tea gardens. The difference of the chemical composition of SOC were primarily represented by alkyl C and aryl C, indicating that alkyl C and aryl C were more liable to change following vegetation changes. The relative abundance of alkyl C was significantly higher, and aryl C was considerably lower in mixed forests than the other vegetation types. The physicochemical characteristics of the soil, as well as tree species diversity were correlated to SOC chemical composition, indicating that they contributed to SOC chemical compositional shifts associated with the vegetation changes. Our results suggest that the conversion of native broadleaved evergreen forests to the other vegetation types following disturbances of short durations and succession may shift the equilibrium states of SOC composition to a different degree, which may potentially drive alterations in SOC and other nutrient cycling in these ecosystems.

## Methods

### Study area

This study was conducted at the Fengyang Mountain nature reserve, Zhejiang Province, China (119°06′ E to 119°15′E, 27°46′ N to 27°58′ N, 600 m to 1929 m a.s.l.), which comprises an area of 15,171 ha. The nature reserve is characterized as a humid subtropical climate with ~2,400 mm of annual rainfall, and an average annual temperature of 12.3 °C. Prior to 1970, this area was dominated by native evergreen broadleaved forests (composed mainly of *Camellia japonica* Linn., *Cyclobalanopsis multiervis* W. C. Cheng et T. Hong, *Schima superba* Gardn. et Champ., *Eurya japonica* Thunb., and *Rhododendron simsii* Planch.). From 1971 to 1973, intensive selective deforestation and reforestation was conducted, and portions of the forests were converted to mixed evergreen broadleaved and coniferous forests (composed primarily of *Schima superba* Gardn. et Champ., *Pinus taiwanensis* Hayata, *Camellia japonica* Linn. and *Eurya japonica* Thunb.) and tea gardens (Table 1). Additionally, some were planted as pure conifer, such as *Cunninghamia lanceolata* (Lamb.) Hook., *Cryptomeria fortune* Hooibrenk ex Otto et Dietr., and bamboo plantations at different elevation following complete clearance. Subsequent to the establishment of the nature reserve in 1975, the entire study area, including the tea gardens has been protected from anthropogenic disturbances. There has since been no fire or insect infestation disturbances recorded as yet. An overview of the main vegetation composition of this study area is presented in Supplementary Information Table S1.

### Sampling

In June 2013, we randomly sampled nine native evergreen broadleaved forest stands, six mixed-forest stands, and four tea garden stands. All sample stands were located on well-drained and mesic sites with slopes of less than 5% to minimize the site effects in soil characteristics^37^,^38^. In each stand, we established a sample plot of 20 × 20 m^2^, in which all trees >2 cm in diameter at breast height (DBH, 1.3 m above root collar) were identified and counted. In each tea garden stand, the tea bush clusters were counted as the quantity of tea bush “individuals”.

Four sampling points were randomly determined within each plot, where sampling points were at least 8 m apart. At each sampling point, we collected soil samples for nuclear magnetic resonance (NMR) spectroscopy, pH, total organic carbon concentration, total nitrogen concentration, and total phosphorus concentration analysis, using a sharp knife and a trowel at the depth from 0–10, 10–20, 20–30 cm after removing litter. For bulk density soil analysis, we carefully extracted soil samples with a metal corer at four randomly allocated sampling point (diameter: 5.5 cm, height: 5 cm)^39^. Samples for nuclear magnetic resonance (NMR) spectroscopy analysis were stored at −80 °C in laboratory until the analysis was conducted^40^.

### Soil physicochemical properties analysis

Soil physicochemical properties of each plot were determined by the mean value of three layers (0–10, 10–20, 20–30 cm) of the four sampling points. Soil bulk density was determined by drying the samples in an oven at 105 °C until a constant weight was achieved, which was then corrected for root and stone volume^38^. Soil samples for other physicochemical analyses were air-dried in the laboratory and then sieved (2 mm mesh) and stored in air-tight plastic bags. Soil pH was measured using a PB-10 pH meter (Sartorius GmbH, Göttingen, Germany) at a 1:5 ratio of soil to water^41^. The total organic carbon concentration was determined using the sulfuric acid-potassium external heating method^42^. Total nitrogen and phosphorus concentrations were determined using a Bran+Luebbe Autoanalyser 3 Continuous Flow Analyzer (Bran+Luebbe GmbH, Norderstedt, Germany) according to the manufacturer’s instructions.

### Nuclear magnetic resonance (NMR) analysis

The ^13^C NMR spectroscopy was conducted on composite samples, which were obtained by mixing three layers and four sampling points from each sampling plot for analysis^14^. Similar to physicochemical analysis, the mixed samples were air-dried in the laboratory and then sieved (2 mm mesh) and stored in air-tight plastic bags in a refrigerator at 4 °C for further investigation^43^. To enhance the signal to noise ratio of the instrument, the hydrofluoric acid (HF) pretreatment was performed to remove Fe^3+^ and Mn^2+^ from the soil^44^,^45^. Samples were processed according to previously published methods^46^. Briefly, 5 g of air-dried soil samples were weighed and transferred into a 100 ml plastic centrifuge tube for the HF pretreatment, followed by the addition of 50 ml HF solution (10% v/v) into the tube. Following vibratory agitation for 1 h and centrifugation for 10 min. (3000 r min^−1^), the supernatant liquid was removed from the tube, and the residue was continuously treated with a HF solution. These steps were repeated 8 times, but varied in the vibration time (4 × 1 h, 3 × 12 h, 1 × 24 h). Soil samples were rinsed 4 times with double distilled water to remove any residual HF that remained. Subsequently, the residues were dried in oven at 40 °C. Following all of the above processes, the residues were ground to the extent that they could pass through a 60-mesh screen, and then loaded into a bag for NMR measurement. The HF-treated soil samples were subjected to ^13^C NMR analysis with a Bruker (Spectrospin, Rheinstetten, Germany) Avance 600 MHz NMR spectrometer. The experiments were carried out using a 7 mm CPMAS probe, at a carbon frequency of 150 MHz, MAS spinning frequency at 6 kHz, contact time of 2 ms, and a recycle delay time of 5 s.

Similar to previous NMR studies^40^,^47^, the NMR spectra were divided into the following regions: alkyl C region (0–45 ppm), N-alkyl C region (45–60 ppm), carbohydrate C region (60–90 ppm), di-O-alkyl C region (90–110 ppm), aryl C region (110–145 ppm), phenolic region (145–165 ppm), carboxyl C region (165–215 ppm). The relative intensities of the different SOC fractions were obtained through the measurement and integration of the area under the curve for each region^46^. Additionally, two indices of organic carbon stability were calculated: the ratio of alkyl C to O-alkyl C (A/O-A) =C_0–45 ppm_/C_45–110 ppm_, which is used as an indicator of organic carbon decomposition potential^48^,^49^, and aromaticity = C_110–165 ppm_/C_0–165 ppm_, which has been used to determine the enrichment in aromatic functional groups in SOC as an indicator of the advanced stages of decomposition^46^,^50^.

### Data analysis

We quantified plant species diversity by employing the Shannon’s index (*H′*):


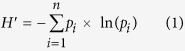


where *p*_*i*_ is the proportion of the individuals of *i*th overstory species in the plot based on stem counts, and *n* is the number of overstory species in the plot^51^, and the index was calculated for each sampling plot. To examine the impacts of vegetation changes on the Shannon index of overstory plants, soil physicochemical characteristics and each SOC chemical composition, one-way analysis of variance (ANOVA) was conducted, followed by a LSD test (*P* < 0.05).

Multiple-response permutation procedures analysis (MRPP) was performed to test the difference of SOC chemical composition of among three vegetation types. The relationship between SOC chemical composition, tree diversity, and soil physicochemical characteristics was analyzed by non-multidimensional scaling (NMDS). Pearson correlation tests were also applied to explore the impacts of the chemical composition of SOC, tree diversity, and soil physicochemical characteristics variables on SOC chemical composition assemblages of NMDS axes. This was achieved by calculating Pearson correlation coefficients between all of the variables and NMDS axes scores. To examine the chemical compositions of SOC, tree diversity, and soil physicochemical characteristics variables, which influenced the chemical composition of SOC assemblages, linear regressions test were performed. To further discover the potential impact of tree diversity and soil physicochemical characteristics on SOC chemical composition, Pearson correlations analysis was also conducted between all components of SOC chemical composition, tree species diversity, and soil physicochemical characteristics. All analyses were performed in R (vegan package)^52^.

## Additional Information

**How to cite this article**: Guo, X. *et al*. Vegetation change impacts on soil organic carbon chemical composition in subtropical forests. *Sci. Rep.* **6**, 29607; doi: 10.1038/srep29607 (2016).

## Supplementary Material

Supplementary Information

## Figures and Tables

**Table 1:** Species diversity index and physicochemical soil characteristics of sampling stands.

Characteristic	Native forest	Mixed forest	Tea garden
Shannon’s index (400 m^2^)	1.61 (0.27)a	0.81 (0.28)b	0.75 (0.17)c
Bulk density (g·cm^−3^)	1.35 (0.06)a	1.35 (0.06)a	1.27 (0.05)b
pH	5.54 (0.26)a	5.24 (0.30)a	5.18 (.034)a
Total organic carbon (g·kg^−1^)	34.90 (8.72)a	30.76 (3.34)a	28.19 (3.26)a
Total nitrogen concentration (g·kg^−1^)	3.16 (0.84)a	3.32 (0.76)a	2.40 (0.70)a
Total phosphorus concentration (g·kg^−1^)	0.49 (0.07)a	0.40 (0.07)b	0.55 (0.05)a

Values shown are means and 1 SE (in bracket). Different letters in the same row indicate a significant difference (*P* < 0.05) between vegetation types.

**Table 2:** Results of multiple-response permutation procedures (MRPP) testing the null hypothesis of no significant differences in the organic carbon chemical composition of the soil among three vegetation types.

Vegetation	Delta	*n*
Native forests	7.858	9
Mixed forests	4.772	6
Tea gardens	8.193	4

Observed delta = 6.954, expected delta = 8.363, chance-corrected within-group agreement, A = 0.169, *P* = 0.003.

**Table 3:** Analysis of variance results for the soil organic carbon chemical composition among three vegetation types.

Composition	df	*F*	*P*
Alkyl C	2, 16	16.460	<0.001
N-alkyl C	2, 16	1.496	0.254
Carbohydrate C	2, 16	0.500	0.616
Di-O-alkyl C	2, 16	0.192	0.827
Aryl C	2, 16	2.808	0.090
Phenolic C	2, 16	1.822	0.194
Carboxyl C	2, 16	2.057	0.160
Alkyl C: O-alkyl C	2, 16	8.824	0.003
Aromaticity	2, 16	2.654	0.101

**Table 4:** Correlation matrix of associations between NMDS axes scores and organic carbon chemical composition for three vegetation types.

Composition	NMDS 1	*P*	NMDS 2	*P*
Alkyl C	−0.618	<0.001	−0.786	<0.001
N-alkyl C	−0.997	<0.001	0.073	0.844
Carbohydrate C	−0.478	<0.001	0.878	<0.001
Di-O-alkyl C	−0.412	<0.001	0.911	<0.001
Aryl C	0.993	<0.001	0.119	0.294
Phenolic C	0.975	<0.001	−0.222	0.379
Carboxyl C	0.840	<0.001	−0.543	0.092
Alkyl C: O-alkyl C	−0.190	<0.001	−0.982	<0.001
Aromaticity	0.997	<0.001	−0.072	0.330

**Table 5:** Correlation matrix of associations between axes scores, tree diversity, and soil physicochemical characteristics for three vegetation types.

Characteristic	NMDS 1	*P*	NMDS 2	*P*
Shannon index	0.973	0.059	0.230	0.778
Bulk density (g·cm^−3^)	−0.977	0.137	0.214	0.840
pH	0.702	0.098	0.713	0.302
Total organic carbon (g·kg^−1^)	−0.293	0.605	0.956	0.319
Total nitrogen concentration (g·kg^−1^)	−0.196	0.548	−0.981	0.085
Total phosphorus concentration (g·kg^−1^)	0.103	0.769	0.995	0.104

**Table 6:** Correlations between SOC chemical composition, soil physicochemical characteristics and Shannon’s index.

Composition	Bulk density		pH		Total carbon concentration		Total nitrogen concentration		Total phosphorus concentration		Shannon’s index	
	*Cor.*	*P*	*Cor.*	*P*	*Cor.*	*P*	*Cor.*	*P*	*Cor.*	*P*	*Cor.*	*P*
Alkyl C	0.251	0.299	−0.469*	**0.043**	−0.025	0.918	0.138	0.574	−0.326	0.173	−0.430	0.065
N-alkyl C	0.336	0.16	−0.476*	**0.039**	−0.023	0.925	0.209	0.389	−0.271	0.262	−0.137	0.577
Carbohydrate C	0.233	0.337	−0.078	0.751	0.21	0.389	0.1	0.683	0.313	0.192	−0.309	0.201
Di-O-alkyl C	0.258	0.286	0.055	0.824	0.3	0.213	0.101	0.682	0.175	0.474	−0.219	0.375
Aryl C	−0.355	0.136	0.405	0.085	−0.202	0.407	−0.209	0.39	0.014	0.955	0.362	0.127
Phenolic C	−0.173	0.48	0.297	0.216	−0.084	0.734	−0.156	0.522	−0.249	0.304	0.536*	**0.018**
Carboxyl C	−0.291	0.227	0.232	0.34	−0.013	0.958	−0.069	0.78	0.219	0.368	0.418	0.077
Alkyl C: O-alkyl C	0.081	0.741	−0.388	0.101	−0.112	0.648	0.034	0.89	−0.396	0.093	−0.210	0.382
Aromaticity	−0.324	0.176	0.37	0.119	−0.157	0.521	−0.202	0.407	−0.018	0.942	0.456*	**0.05**

^*^Correlation is significant at *P* < 0.05.
