# Supplementary material for: Vegetation change impacts on soil organic carbon chemical composition in subtropical forests
Source: Sci Rep. 2016 Jul 11;6:29607. doi: 10.1038/srep29607 (PMC4939599; doi:10.1038/srep29607)
Supplement: Supplementary Information [file srep29607-s1.doc]

# Stand type change impacts on soil organic carbon chemical composition in subtropical forests

Xiaoping Guo^1, 2^, Miaojing Meng^1^, Jinchi Zhang^1,*^, Han Y. H. Chen^2,*^

^1^Collaborative Innovation Center of Sustainable Forestry in Southern China of Jiangsu Province, Nanjing Forestry University, 159 Longpan Road, Nanjing, Jiangsu 210037, China

^2^Faculty of Natural Resource Management, Lakehead University, 955 Oliver Road, Thunder Bay, Ontario P7B 5E1, Canada

Address correspondence*

*Corresponding authors: email: zhang8811@njfu.edu.cn (Jinchi Zhang), phone & fax: +86-25-8542-7471; email: [hchen1@lakeheadu.ca](mailto:hchen1@lakeheadu.ca) (Han Y. H. Chen), phone: +1-807-343-8342, fax: +1-807- 343-8116.

# Supplementary Information

### *Table S1 Characteristics of forest stands sampled*

| Vegetation type | Stand Age (years) | Density (stems/ha)* | Main species and composition (%)* |
| --- | --- | --- | --- |
| Evergreen broadleaved forest | Old-growth | 536 (105)b | *Camellia japonica* [18 (15)], *Cyclobalanopsis multiervis* [18 (17)], *Schima superba* [8 (12)], *Eurya japonica* [3 (4)], *Rhododendron simsii* [8 (10)] and others [45 (22)] |
| Mixed forest | 43 | 437 (72)b | *Pinus taiwanensis* [13 (8)], *Schima superba* [53 (30)], *Camellia japonica*[6 (14)], *Eurya japonica*[5 (6)], and others [23 (32)] |
| Tea garden | 42 | 1200 (68)a | *Tea* [92 (4)] and others [8 (4)] |

*Mean and one standard deviation in parentheses.
